# Incidence of postoperative administration of opioids in dogs undergoing a tibial plateau leveling osteotomy after intra-operative liposomal bupivacaine administration with or without morphine epidural

**DOI:** 10.1186/s12917-023-03664-7

**Published:** 2023-07-31

**Authors:** Jessie Scaglione, Jacqueline Carver

**Affiliations:** Surgery Department, The Veterinary Medical Center of Long Island, 75 Sunrise Highway, West Islip, NY USA

**Keywords:** TPLO, Nocita, Epidural

## Abstract

**Objective:**

To determine the influence of two intraoperative pain management protocols on the need for additional opioids in patients receiving an epidural and periarticular liposomal bupivacaine (PLB) vs. PLB alone in the 12–24 h period after undergoing a TPLO.

**Study design:**

Retrospective study.

**Animals:**

One hundred seventy-four dogs with cranial cruciate ligament tears presenting for TPLO.

**Methods:**

Medical records of dogs presenting for a unilateral or bilateral cranial cruciate ligament tear who had a TPLO performed were reviewed for signalment, weight, body condition score, and peri-operative pain management protocol. Dogs were divided into two groups: those who received an epidural and PLB, and those who received only PLB. Post-operative opioid administration was recorded for each group.

**Results:**

Patients who received an epidural and PLB received fewer postoperative opioids. There were 36% fewer opioid injections administered to dogs who received epidurals compared to dogs who did not receive epidurals (IRR) (95% CI) = 0.64 (0.45–0.92), *P* = 0.02). BCS was not a significant predictor of the post-operative opioid requirement (IRR (95% CI) = 1.3 (0.75–2.4), *P* = 0.38). When adjusting for BCS as a possible confounder, there were 39% fewer opioid injections in dogs who received epidurals than dogs without (IRR (95% CI) = 0.61 (0.42–0.88), *P* = 0.009).

**Conclusions:**

The incidence of postoperative opioid administration was significantly diminished in patients receiving both an epidural and PLB.

**Clinical significance:**

Administration of an epidural in addition to infiltration of PLB significantly decreased the incidence of postoperative opioid administration in dogs undergoing a TPLO.

**Supplementary Information:**

The online version contains supplementary material available at 10.1186/s12917-023-03664-7.

## Introduction

Cranial cruciate ligament (CrCL) injury is recognized as one of the most common causes of pelvic limb lameness in dogs [1–6]. Persistent instability leading to abnormal dynamic joint function is one of the causes of osteoarthritis in the CrCL deficient stifle [4, 7–9]. Although the exact mechanisms are still unclear, it is generally accepted that CrCL disease in dogs is a degenerative process. Some dogs will present with acute ruptures secondary to acute trauma such as excessive limb loading, hyperextension, and excessive internal rotation. While some patients are managed conservatively, surgical stabilization is generally the primary recommendation. The goal of surgery is to neutralize the tibiofemoral shear force and restore normal range of motion and function. There are multiple procedures described, and of those, the tibial plateau leveling osteotomy (TPLO) is often favored. The TPLO continues to produce predictable outcomes with a stable construct that allows for immediate weight bearing [1–3, 10, 11].

Perioperative pain management must be considered for patients presenting for TPLO. Frequently used analgesics include opioids, alpha-2 agonists, ketamine, non-steroidal anti-inflammatories, and local anesthetics. Of those, opioids tend to be favored because they are safe and effective, but not all of the effects of opioids are desirable. They often cause dysphoria, gastrointestinal upset, decrease gastrointestinal motility, and can cause profound sedation at times [1, 12]. Alternatives to opioid administration include local anesthetics and epidural analgesia, especially since IV administration of opioids is often less effective in managing post-operative pain when compared to epidural analgesia and/or nerve blocks [12].

Epidural administration of opioids and local anesthetics are employed to prevent central sensitization to pain by mitigating the nociceptive input to the dorsal horn of the spinal cord at the level of the nerve root [13]. Not only do epidurals provide effective pain control postoperatively, they also are associated with fewer adverse effects that are appreciated when the same drug is given IV [8, 13].

A liposomal form of bupivacaine (Nocita, Aratana Therapeutics, Leawood, Kansas) was initially introduced and approved for use in dogs undergoing stifle surgery. It is composed of an aqueous form of bupivacaine surrounded by multivesicular liposomes, which are made up of nonconcentric lipid bilayers. Liposomal bupivacaine is reported to provide post-operative pain relief for up to 72 h [14, 15]. During those 72 h, enzymes slowly break down the bilayers, releasing bupivacaine into surrounding tissues. Liposomal bupivacaine is a widely used local anesthetic that is a component of a multimodal pain management protocol with the potential to minimize the need for postoperative opioids [14, 16]. The objective of this study was to compare the incidence of postoperative opioid administration in patients undergoing a TPLO who received periarticular liposomal bupivacaine (PLB) alone, and patients who received both an epidural and PLB. We hypothesized that those receiving both an epidural and PLB would receive fewer doses of postoperative opioids. To the authors' knowledge, no studies have evaluated the incidence for postoperative opioid in dogs undergoing a TPLO who received PLB and an epidural vs. PLB alone.

## Materials and methods

### Inclusion and exclusion criteria

A search of the medical records databases at The Veterinary Medical Center of Long Island was performed for dogs that underwent a TPLO for treatment of a CrCL tear between 2017 and 2021. A total of 204 dogs had a TPLO performed during that time. Dogs were included if there was documentation of epidural administration, PLB, and post-operative opioid administration. Dogs were excluded if they were placed on a Fentanyl CRI postoperatively, if they had additional procedures performed (ex. PRP injection in the contralateral stifle, excisional biopsy), or if the medical records were incomplete. A total of 174 patients were included. Of the stifles, 26 were bilateral and 148 were unilateral. Patients who had bilateral TPLOs were staged at least 3 weeks between procedures.

### Data collection

Data retrieved included age, body weight, body condition score, gender and reproductive status, breed, limb operated, the number of postoperative opioid injections, and whether the patient received an epidural, PLB, or both. All procedures were performed by a board-certified veterinary surgeon (JC). Patients were divided into two groups based on whether they received an epidural and PLB, or PLB without epidural.

All patients were premedicated with either Morphine (0.5 mg/kg IM or IV) or Buprenorphine (0.015–0.02 mg/kg IM or IV), and either acepromazine (0.01–0.02 mg/kg IM or IV) or Midazolam (0.2 mg/kg IV or IM). Patients were induced with propofol (4–6 mg/kg IV to effect), intubated, and maintained on Isoflurane and oxygen. Epidurals consisted of either preservative free morphine (0.1 mg/kg) alone, or bupivacaine (0.5 mg/kg). Epidural drug choice was primarily influenced by which drug was readily available at that time. All patients received installation of periarticular PLB (5.3 mg/kg) in the subcutaneous and intradermal layers during closure. In most instances, PLB was not diluted. When it was diluted, it was diluted to no more than 1:1 with sterile saline. Postoperatively, all patients were assessed every 6 to 8 h. Patients were assessed by either an emergency clinician or a surgeon prior to administration of either Morphine or Buprenorphine using the Colorado State-University-Canine Acute Pain Scale (CSU-CAPS). Utilizing this pain scale was useful in that it provided the clinician assessing the patient with objective criteria with which to determine if a patient was in need of rescue analgesia. Post-operative (PO) doses of opioids were the same as premedication doses. The decision to withhold opioids was made when the patient was alert but calm, non-reactive, willing to move in and out of the cage with assistance, bearing partial or full weight on the surgical limb, and when the patient was willing to eat and take oral medications. The decision to administer opioids was made when the patient was vocalizing, reactive when they were taking in and out of the cage, guarding the limb, showed signs of aggression, unwilling to interact with staff, and was unwilling to eat.

The majority of patients were discharged from the hospital within 12–24 h of surgery, however, ongoing pain management in the form of opioids was considered for those remaining in hospital for up to 48 h. Although not utilized until 24 h PO, non-steroidal anti-inflammatory drugs (NSAIDs) are commonly used peri-operatively. The patients in the present study were administered NSAIDs orally the morning following surgery. A modification to our current protocol could be the administration of an NSAID SQ immediately PO. All patients were re-evaluated at 14 days for routine recheck exam, and at 10–12 weeks postoperatively for sedated radiographs to ensure complete healing of the osteotomy site.

### Statistical analysis

Data were analyzed using SAS 9.4 (Cary, NC). When comparing patients who did and did not receive an epidural, a student t-test was used for age and weight. Fischer’s exact test was used to determine associations between categorical variables, which included sex and breed, and side operated (left vs. right pelvic limb) was evaluated using a chi-square test. A multivariable negative binomial regression using epidural as a covariant of body condition scores was developed to account for BCS as a possible source of bias or confounding. Breeds with less than 5 animals were grouped together in an Other Breed category. Continuous data were recorded as mean ± SD. *P* < 0.05 was considered significant.

## Results

One hundred seventy-four patients who underwent a TPLO met the inclusion criteria between December 2017 and January 2021. All TPLO procedures were performed by a single boarded surgeon (JC). The mean age of the dogs included was 5.9 ± 2.4 years, and the mean body weight was 33.1 ± 11.2 kgs. Of the 174 dogs included, 40.23% (70/174) received no PO opioid injections, 35.63% (62/174) received 1 injection, 18.97% (33/174) received 2 injections, 3.34% (6/174) received 3 opioid injections, 0.57% (1/174) received 4 opioid injections, and 1.15% (2/174) received 6 opioid injections (Table 1). There was 1 dog with a BCS score of 2, 153 dogs with a BCS score of 3, 4 dogs with a BCS score of 4, and 1 dog with a missing BCS score out of 174 dogs.

**Table 1:** Distribution of number of PO opioid injections by epidural use

# of PO Opioid Injections	Epidural (*n* = 137) n(%)	No Epidural (*n* = 37) n(%)
0	59 (43%)	11 (30%)
1	51 (37%)	11 (30%)
2	22 (16%)	11 (30%)
3	3 (2%)	3 (8%)
4	1 (1%)	0 (0%)
6	1 (1%)	1 (3%)

Abbreviations: *PO* Post-operative

**Fig. 1:**
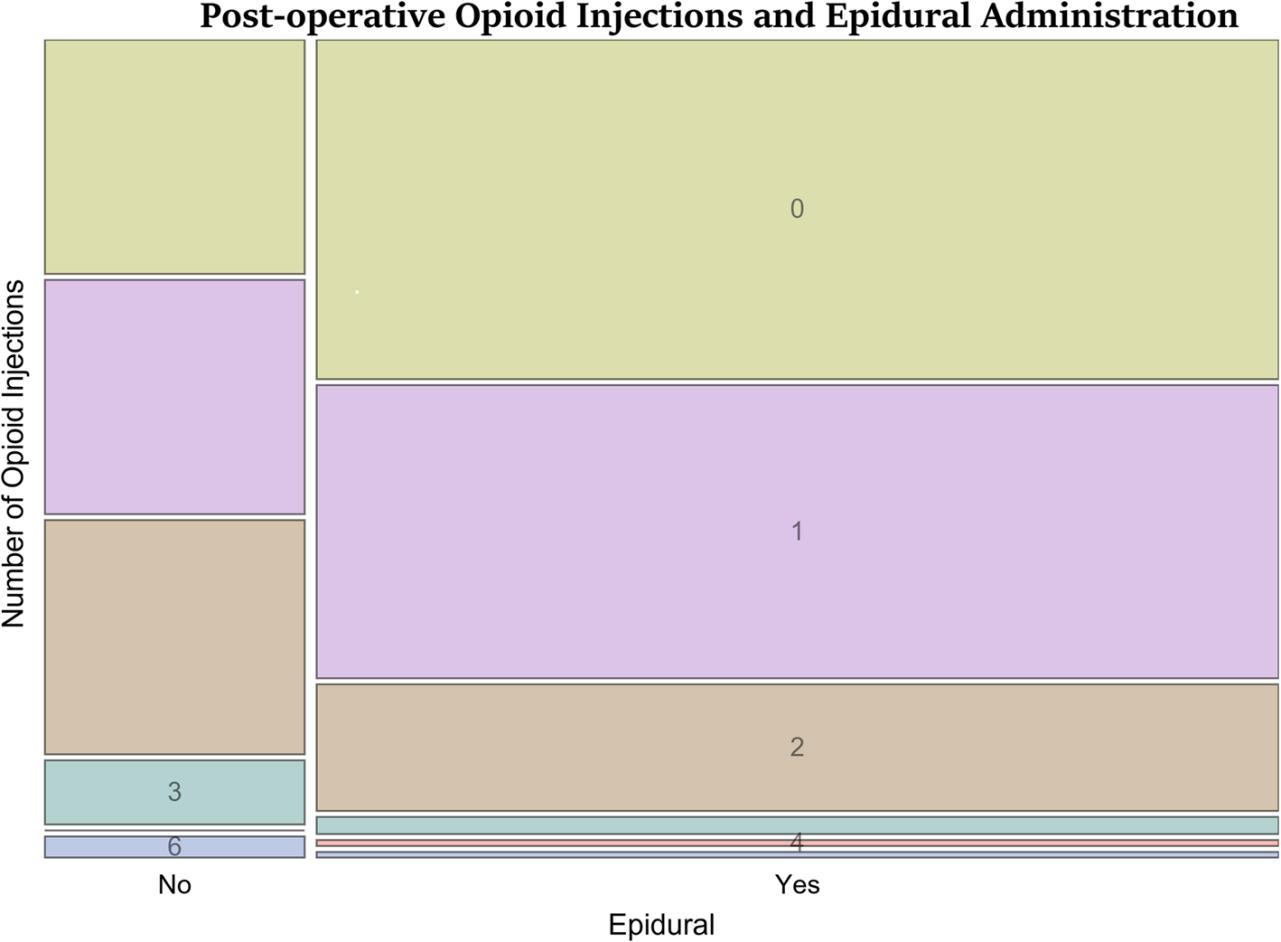
Distribution of the number of opioid injections given post-operatively. Patients were divided into those who received an epidural and those who received no epidural. Patients who received both an epidural and PLB were administered fewer post-operative opioid injections

The primary comparison of the number of opioid injections as the dependent variable and epidural as a predictive factor was performed using negative binomial regression. There were 36% fewer opioid injections administered to dogs who received epidurals than dogs who did not receive epidurals (IRR) (95% CI) = 0.64 (0.45–0.92), *P* = 0.02) (Fig. 1). BCS was not a significant predictor of the number of opioid injections administered to a patient PO (IRR (95% CI) = 1.3 (0.75–2.4), *P* = 0.38). When adjusting for BCS as a possible confounder, there were 39% fewer opioid injections in dogs with epidurals than dogs without (IRR (95% CI) = 0.61 (0.42–0.88), *P* = 0.009).

The proportion of dogs who received epidurals ((11/136) 8%) that had BCS of 4/5 was lower than the proportion of dogs who did not receive epidurals ((7/37) 22%) that had a BCS of 4/5 (*P* = 0.03). The mean ± SD of weight and age of dogs given epidurals was 33 ± 11 kg and 5.9 ± 2.3 years respectively. The mean ± SD of weight and age of dogs not given epidurals was 32 ± 13 kg (*P* = 0.41) and 6.2 ± 2.8 years (*P* = 0.52) respectively. The distribution of breeds (*P *= 0.31), sex (*P* = 0.31) and limb operated (*P* = 0.45) were not significantly different between dogs who did and did not receive epidurals.

## Discussion

Patients undergoing a TPLO benefit from a multimodal pain management protocol. There is overwhelming evidence that patients who receive opioids in combination with epidurals, peripheral nerve blocks, or administration of intra-articular opioids consistently demonstrate that they are more comfortable in the PO period [1, 13, 17–22]. In the present study, dogs undergoing a TPLO for a ruptured CrCL who received both an epidural and PLB had a significantly diminished incidence of PO opioid injections when compared to dogs who received PLB alone. Our hypothesis was accepted, as 36% fewer PO opioid injections were administered to patients who received both an epidural and PLB. Breed, sex, and limb operated were not significantly different between dogs who did and did not receive an epidural, and BCS was not indicative of the number of opioid injections administered PO. Those receiving an epidural did not demonstrate urine retention.

The majority of patients included were given zero to two PO opioid injections, supporting the practice of a multimodal pain management protocol. Some of the benefits of a protocol that minimizes the administration of post-operative IV opioids are fewer incidences of dysphoria and decreased gastrointestinal upset [14, 21–23]. Bini et al. demonstrated that patients who were administered methadone every 4 h despite their pain score were 38% less likely to eat than those given methadone in response to a moderate to high pain score [21]. Anecdotally, patients in the current study appear more likely to eat shortly after surgery. This allows earlier transition to oral pain medications. Additionally, minimizing the number of PO opioid injections also has the ability to minimize the financial burden on the client through earlier discharge. Lastly, patients demonstrate an earlier return to ambulation [1].

Of the dogs included, there were several patients who received three or more injections of opioids PO. Of those, 4% (5/137) received both an epidural and PLB, and 11% (4/37) received PLB alone. It is possible that the patient's overall demeanor contributed to the administration of additional opioid injections. We recognize that some patients are anxious in the hospital environment, and may have received an opioid with or without acepromazine for anxiety, even if they were not assessed as painful. Therefore, it is possible that anxiety was a confounding factor in pain assessment, and may have led to potentially unnecessary opioid administration.

Epidural administration of local anesthetics and opioids is a well described and widely accepted method of analgesia for dogs undergoing stifle surgery [1, 24]. The combination of an epidural and infiltration of a long-acting anesthetic allows the patient to remain comfortable with a consistent and reliable source analgesia. However, as with any procedure, epidurals are not without potential complications. Documented complications associated with epidurals include, but are not limited to infection, hematoma formation, urine retention, regional vasodilation, and damage to the spinal cord and/or nerve roots [20]. An alternative to epidural analgesia are nerve blocks, which can also be combined with PLB.

In both humans and veterinary patients, peripheral nerve blocks provide local analgesia without some of the potential undesirable complications associated with epidural administration [7, 13, 20]. They prevent neuronal impulses from reaching the spinal cord at the level of the proximal peripheral nerve [7, 19]. McCally et al. evaluated the difference between patients receiving a femoral nerve block (FNB), a FNB in combination with a sciatic nerve block (SCN), and an epidural in dogs who were undergoing a unilateral TPLO. They found that there was not a significant difference in PO pain control between peripheral nerve blocks and an epidural. They did, however, determine that patients who only received a FNB did have higher pain scores, indicating that FNB in combination with SNB is preferable [19]. Regardless of the combination of analgesic techniques, our study is in agreement with previous studies that support the use of a multimodal pain management protocol. While the focus of this study was to assess the need for PO opioid administration in those receiving an epidural and PLB vs. PLB alone, future studies could evaluate a population of patients who receive only an epidural. The objective being to assess if PLB is unnecessary when an epidural is provided.

The study presented has several limitations, the main limitation being its retrospective nature. Additionally, pain assessment has long been difficult to assess in veterinary patients [19]. The patients in this study were assessed by either an emergency clinician or a surgeon prior to opioid administration. The Colorado State-University-Canine Acute Pain Scale was utilized to assess pain in all patients. Future studies evaluating administration of anxiolytics prior to recovery may help decrease the impact that anxiety has on PO opioid administration.

Additional limitations included the small sample size in the no epidural group. The majority of patients undergoing a TPLO at our facility receive an epidural. The most common reason why a patient would not receive an epidural is obesity, which can make it difficult to identify landmarks. Finally, neither the surgeon nor emergency clinician were blinded to the epidural injection. This does allow for bias when assessing patients.

In conclusion, the results of this study demonstrated a significant decrease in administration of PO opioids when epidural and PLB were used in combination. The findings in this study support the use of a multimodal pain management protocol that incorporates local and regional analgesia. This work emphasizes the importance of optimizing pain management protocols for post-operative patients, and highlights the need for future analgesia studies.

## Supplementary Information

**Additional file 1. **Statistical analysis of nocita vs Nocita+Epidural on number of opioid injections in TPLO surgeries.

## Data Availability

All data generated and analyzed during the current study have been included as Supplementary files. Additional information and/or explanation of data is available upon request should the raw data supplied not be satisfactory. Please contact Jessie Scaglione (Jscaglione@vmcli.com).
