# Supplementary material for: Incidence of postoperative administration of opioids in dogs undergoing a tibial plateau leveling osteotomy after intra-operative liposomal bupivacaine administration with or without morphine epidural
Source: BMC Vet Res. 2023 Jul 31;19:102. doi: 10.1186/s12917-023-03664-7 (PMC10388522; doi:10.1186/s12917-023-03664-7)
Supplement: Supplementary file 1 — Additional file 1. Statistical analysis of nocita vs Nocita+Epidural on number of opioid injections in TPLO surgeries. [file 12917_2023_3664_MOESM1_ESM.docx]

**Statistical Analysis of Nocita vs Nocita+Epidural on Number of Opioid Injections in TPLO surgeries**

Requested by: Dr. Jessie Scaglione, DVM

Consulting Statistician: Deborah A. Keys, Ph.D.

The study was an observational retrospective study.

**Animals:** 174 dogs getting TPLO surgery and either Nocita or Nocita + epidural

**Data:** endpoint=# of opioid injections, independent = Nocita or Nocita+epi, general population info: age, weight, sex, breed, limb, BCS, potential confounder=BCS

**Statistical Analysis:** All analyses were performed using SAS 9.4 (Cary, NC). A significance threshold of 0.05 was used.

Histograms and QQ plots confirmed age and weight data were approximately normally distributed and were summarized as Mean ± SD. The primary comparison of number of opioid injections as the dependent variable and epidural as a predictive factor was performed using negative binomial regression. Comparisons between dogs that did and did not receive epidurals were made for age and weight with student t-tests, for body condition scores ((2 or 3) vs 4), sex and breed distributions with Fisher’s exact tests and side by a chi-square test. Breeds with less than 5 animals were grouped together in an Other Breed category. A multivariable negative binomial regression with epidural and a covariate of body condition scores was developed to account for body condition scores as a possible source of bias or confounding.

**Results:**

***General Population***

Mean ± SD age was 5.9 + 2.4 years and weight was 33.1 ± 11.2 kg for all dogs. Median (IQR) 1 (0,1)

| *Variable* | *N* | *Mean* | *Std Dev* | *Median* | *Lower Quartile* | *Upper Quartile* | *Minimum* | *Maximum* |
| --- | --- | --- | --- | --- | --- | --- | --- | --- |
| Weight (kgs) Age (years) #PO opiod injections BCS | 171 168 174 173 | 33.1 5.9 0.9 3.1 | 11.2 2.4 1.0 0.3 | 32.7 6.0 1.0 3.0 | 26.3 4.0 0.0 3.0 | 39.5 7.5 1.0 3.0 | 5.2 1.0 0.0 2.0 | 66.8 11.5 6.0 4.0 |

There were 70 dogs with 0 opioid injections, 62 dogs with 1 opioid injections, 33 with 2 opioid injections, 6 with 3 opioid injections, 1 with 4 opioid injections and 2 with 6 opioid injections out of 174 total dogs. There was 1 dog with a BCS score of 2, 153 dogs with a BCS score of 3 and 4 dogs with a BCS score of 4 and 1 dog with a missing BCS score out of 174 dogs.

| *PO opiod injections* | | | | |
| --- | --- | --- | --- | --- |
| *PO_opiod_injections* | *Frequency* | *Percent* | *Cumulative Frequency* | *Cumulative Percent* |
| *0* | 70 | 40.23 | 70 | 40.23 |
| *1* | 62 | 35.63 | 132 | 75.86 |
| *2* | 33 | 18.97 | 165 | 94.83 |
| *3* | 6 | 3.45 | 171 | 98.28 |
| *4* | 1 | 0.57 | 172 | 98.85 |
| *6* | 2 | 1.15 | 174 | 100.00 |

| *BCS* | | | | |
| --- | --- | --- | --- | --- |
| *BCS* | *Frequency* | *Percent* | *Cumulative Frequency* | *Cumulative Percent* |
| *2* | 1 | 0.58 | 1 | 0.58 |
| *3* | 153 | 88.44 | 154 | 89.02 |
| *4* | 19 | 10.98 | 173 | 100.00 |
| *Frequency Missing = 1* | | | | |

| *Breed* | | | | |
| --- | --- | --- | --- | --- |
| *Breed* | *Frequency* | *Percent* | *Cumulative Frequency* | *Cumulative Percent* |
| *Alapaha Bulldog* | 2 | 1.16 | 2 | 1.16 |
| *American Pitbull* | 1 | 0.58 | 3 | 1.74 |
| *American Staffordshire Terrier* | 1 | 0.58 | 4 | 2.33 |
| *Australian Shepherd Mi* | 1 | 0.58 | 5 | 2.91 |
| *Basset Hound* | 1 | 0.58 | 6 | 3.49 |
| *Beagle* | 3 | 1.74 | 9 | 5.23 |
| *Beagle Mix* | 1 | 0.58 | 10 | 5.81 |
| *Bernese Mountain Dog* | 3 | 1.74 | 13 | 7.56 |
| *Boxer* | 4 | 2.33 | 17 | 9.88 |
| *Brittany Spaniel* | 1 | 0.58 | 18 | 10.47 |
| *Bull Mastiff* | 3 | 1.74 | 21 | 12.21 |
| *Bulldog* | 3 | 1.74 | 24 | 13.95 |
| *Cane Corso* | 1 | 0.58 | 25 | 14.53 |
| *Carolina Mix* | 1 | 0.58 | 26 | 15.12 |
| *Cavalier King Charles Spaniel* | 3 | 1.74 | 29 | 16.86 |
| *Chow Mix* | 1 | 0.58 | 30 | 17.44 |
| *Coton De Tulear* | 1 | 0.58 | 31 | 18.02 |
| *Doberman Mix* | 2 | 1.16 | 33 | 19.19 |
| *English Bulldog* | 1 | 0.58 | 34 | 19.77 |
| *German Shepherd Mix* | 1 | 0.58 | 35 | 20.35 |
| *Golden Retriever* | 13 | 7.56 | 48 | 27.91 |
| *Goldendoodle* | 4 | 2.33 | 52 | 30.23 |
| *Great Dane* | 3 | 1.74 | 55 | 31.98 |
| *Great Pyrenes Mix* | 1 | 0.58 | 56 | 32.56 |
| *Hound Mix* | 5 | 2.91 | 61 | 35.47 |
| *Irish Water Spaniel* | 1 | 0.58 | 62 | 36.05 |
| *Labradoodle* | 1 | 0.58 | 63 | 36.63 |
| *Labrador Mix* | 12 | 6.98 | 75 | 43.60 |
| *Labrador Retriever* | 24 | 13.95 | 99 | 57.56 |
| *Miniature Pinscher* | 1 | 0.58 | 100 | 58.14 |
| *Mixed Breed* | 21 | 12.21 | 121 | 70.35 |
| *Morkie* | 1 | 0.58 | 122 | 70.93 |
| *Newfoundland* | 1 | 0.58 | 123 | 71.51 |
| *Pit Bull Mix* | 18 | 10.47 | 141 | 81.98 |
| *Pit Bull Terrier* | 8 | 4.65 | 149 | 86.63 |
| *Rottweiler* | 8 | 4.65 | 157 | 91.28 |
| *Rottweiler Mix* | 1 | 0.58 | 158 | 91.86 |
| *Saint Bernard* | 1 | 0.58 | 159 | 92.44 |
| *Schnauzer* | 1 | 0.58 | 160 | 93.02 |
| *Shepherd Mix* | 2 | 1.16 | 162 | 94.19 |
| *Shiba Inu* | 3 | 1.74 | 165 | 95.93 |
| *Siberian Husky* | 2 | 1.16 | 167 | 97.09 |
| *Soft-coated Wheaten Terrier* | 1 | 0.58 | 168 | 97.67 |
| *Terrier Mix* | 3 | 1.74 | 171 | 99.42 |
| *Wheaten Terrier* | 1 | 0.58 | 172 | 100.00 |
| *Frequency Missing = 2* | | | | |

| *Breed>=5* | *Frequency* | *Percent* | *Cumulative Frequency* | *Cumulative Percent* |
| --- | --- | --- | --- | --- |
| *Golden Retriever* | 13 | 7.47 | 13 | 7.47 |
| *Hound Mix* | 5 | 2.87 | 18 | 10.34 |
| *Labrador Mix* | 12 | 6.90 | 30 | 17.24 |
| *Labrador Retriever* | 24 | 13.79 | 54 | 31.03 |
| *Mixed Breed* | 21 | 12.07 | 75 | 43.10 |
| *Other LT5* | 65 | 37.36 | 140 | 80.46 |
| *Pit Bull Mix* | 18 | 10.34 | 158 | 90.80 |
| *Pit Bull Terrier* | 8 | 4.60 | 166 | 95.40 |
| *Rottweiler* | 8 | 4.60 | 174 | 100.00 |

| *Sex* | | | | |
| --- | --- | --- | --- | --- |
| *Sex* | *Frequency* | *Percent* | *Cumulative Frequency* | *Cumulative Percent* |
| *FI* | 1 | 0.59 | 1 | 0.59 |
| *FS* | 85 | 50.00 | 86 | 50.59 |
| *MI* | 2 | 1.18 | 88 | 51.76 |
| *MN* | 82 | 48.24 | 170 | 100.00 |
| *Frequency Missing = 4* | | | | |

| *Limb* | | | | |
| --- | --- | --- | --- | --- |
| *Limb* | *Frequency* | *Percent* | *Cumulative Frequency* | *Cumulative Percent* |
| *Left* | 84 | 48.55 | 84 | 48.55 |
| *Right* | 89 | 51.45 | 173 | 100.00 |
| *Frequency Missing = 1* | | | | |

**Primary Comparison**

There were 36% less opioid injections in dogs with epidurals than dogs without (incident rate ratio (IRR) (95% CI) = 0.64 (0.45-0.92), p=0.02). BCS was not a significant predictor of number of opioid injections (IRR (95% CI) = 1.3 (0.75-2.4), p=0.38). When adjusting for BCS as a possible confounder, there were 39% less opioid injections in dogs with epidurals than dogs without (IRR (95% CI) = 0.61 (0.42-0.88), p=0.009).

| *Analysis Variable : PO_opiod_injections PO opiod injections* | | | | | | | | | |
| --- | --- | --- | --- | --- | --- | --- | --- | --- | --- |
| *Epidural* | *N Obs* | *N* | *Mean* | *Std Dev* | *Median* | *Lower Quartile* | *Upper Quartile* | *Minimum* | *Maximum* |
| No | 37 | 37 | 1.30 | 1.24 | 1.00 | 0.00 | 2.00 | 0.00 | 6.00 |
| Yes | 137 | 137 | 0.83 | 0.95 | 1.00 | 0.00 | 1.00 | 0.00 | 6.00 |

| *Table of PO_opioid_injections by Epidural* | | | |
| --- | --- | --- | --- |
| *PO_opiod_injections* | *Epidural* | | |
| *Frequency Col Pct* | *No* | *Yes* | *Total* |
| *0* | 11 30% | 59 43% | 70 |
| *1* | 11 30% | 51 37% | 62 |
| *2* | 11 30% | 22 16% | 33 |
| *3* | 3 8% | 3 2% | 6 |
| *4* | 0 0% | 1 1% | 1 |
| *6* | 1 3% | 1 1% | 2 |
| *Total* | 37 | 137 | 174 |

Mosaic Plot of Epidural by Number of Opioid Injections

| *Table of PO_opiod_injections by BCScat* | | | |
| --- | --- | --- | --- |
| *PO_opiod_injections(PO opiod injections)* | *BCScat* | | |
| *Frequency Col Pct* | *2 or 3* | *4* | *Total* |
| *0* | 61 39.61 | 9 47.37 | 70 |
| *1* | 56 36.36 | 6 31.58 | 62 |
| *2* | 28 18.18 | 4 21.05 | 32 |
| *3* | 6 3.90 | 0 0.00 | 6 |
| *4* | 1 0.65 | 0 0.00 | 1 |
| *6* | 2 1.30 | 0 0.00 | 2 |
| *Total* | 154 | 19 | 173 |
| *Frequency Missing = 1* | | | |

**By Epidural**

The proportion of dogs with epidurals ((11/136) 8%) that had BCS of 4/5 was lower than proportion of dogs without epidurals ((7/37) 22%) that had a BCS (p=0.03). The mean + SD weight and age of dogs with epidurals was 33+11 kg and 5.9 + 2.3 years and without epidurals was 32+13 kg (p=0.41) and 6.2 + 2.8 years (p=0.52). The distribution of breeds (p=0.31), sex (p=0.31) and limbs (p=0.45) were not significantly different between dogs that did and did not receive epidurals.

Mosaic Plot of Epidural by BCS

| *Epidural* | *N Obs* | *Variable* | *N* | *Mean* | *Std Dev* | *Median* | *Lower Quartile* | *Upper Quartile* | *Minimum* | *Maximum* | *p-value* |
| --- | --- | --- | --- | --- | --- | --- | --- | --- | --- | --- | --- |
| No | 37 | Weight__kgs_ Age__years_ BCS | 36 36 37 | 31.7 6.2 3.2 | 12.6 2.8 0.4 | 31.1 6.5 3.0 | 24.2 3.8 3.0 | 40.6 8.5 3.0 | 5.2 1.5 3.0 | 66.0 10.5 4.0 | 0.41  0.52  0.03 |
| Yes | 137 | Weight__kgs_ Age__years_ BCS | 135 132 136 | 33.4 5.9 3.1 | 10.9 2.3 0.3 | 33.0 6.0 3.0 | 26.8 4.0 3.0 | 39.3 7.5 3.0 | 5.8 1.0 2.0 | 66.8 11.5 4.0 |  |

| *Table of Breed by Epidural* | | | |
| --- | --- | --- | --- |
| *Breed(Breed)* | *Epidural(Epidural)* | | |
| *Frequency Col Pct* | *No* | *Yes* | *Total* |
| *Alapaha Bulldog* | 1 2.70 | 1 0.74 | 2 |
| *American Pitbull* | 0 0.00 | 1 0.74 | 1 |
| *American Staffordshire Terrier* | 0 0.00 | 1 0.74 | 1 |
| *Australian Shepherd Mi* | 1 2.70 | 0 0.00 | 1 |
| *Basset Hound* | 0 0.00 | 1 0.74 | 1 |
| *Beagle* | 2 5.41 | 1 0.74 | 3 |
| *Beagle Mix* | 0 0.00 | 1 0.74 | 1 |
| *Bernese Mountain Dog* | 0 0.00 | 3 2.22 | 3 |
| *Boxer* | 1 2.70 | 3 2.22 | 4 |
| *Brittany Spaniel* | 0 0.00 | 1 0.74 | 1 |
| *Bull Mastiff* | 0 0.00 | 3 2.22 | 3 |
| *Bulldog* | 0 0.00 | 3 2.22 | 3 |
| *Cane Corso* | 0 0.00 | 1 0.74 | 1 |
| *Carolina Mix* | 0 0.00 | 1 0.74 | 1 |
| *Cavalier King Charles Spaniel* | 1 2.70 | 2 1.48 | 3 |
| *Chow Mix* | 1 2.70 | 0 0.00 | 1 |
| *Coton De Tulear* | 0 0.00 | 1 0.74 | 1 |
| *Doberman Mix* | 0 0.00 | 2 1.48 | 2 |
| *English Bulldog* | 1 2.70 | 0 0.00 | 1 |
| *German Shepherd Mix* | 0 0.00 | 1 0.74 | 1 |
| *Golden Retriever* | 1 2.70 | 12 8.89 | 13 |
| *Goldendoodle* | 1 2.70 | 3 2.22 | 4 |
| *Great Dane* | 1 2.70 | 2 1.48 | 3 |
| *Great Pyrenes Mix* | 0 0.00 | 1 0.74 | 1 |
| *Hound Mix* | 0 0.00 | 5 3.70 | 5 |
| *Irish Water Spaniel* | 0 0.00 | 1 0.74 | 1 |
| *Labradoodle* | 0 0.00 | 1 0.74 | 1 |
| *Labrador Mix* | 0 0.00 | 12 8.89 | 12 |
| *Labrador Retriever* | 8 21.62 | 16 11.85 | 24 |
| *Miniature Pinscher* | 0 0.00 | 1 0.74 | 1 |
| *Mixed Breed* | 6 16.22 | 15 11.11 | 21 |
| *Morkie* | 1 2.70 | 0 0.00 | 1 |
| *Newfoundland* | 0 0.00 | 1 0.74 | 1 |
| *Pit Bull Mix* | 3 8.11 | 15 11.11 | 18 |
| *Pit Bull Terrier* | 2 5.41 | 6 4.44 | 8 |
| *Rottweiler* | 2 5.41 | 6 4.44 | 8 |
| *Rottweiler Mix* | 0 0.00 | 1 0.74 | 1 |
| *Saint Bernard* | 0 0.00 | 1 0.74 | 1 |
| *Schnauzer* | 1 2.70 | 0 0.00 | 1 |
| *Shepherd Mix* | 1 2.70 | 1 0.74 | 2 |
| *Shiba Inu* | 1 2.70 | 2 1.48 | 3 |
| *Siberian Husky* | 1 2.70 | 1 0.74 | 2 |
| *Soft-coated Wheaten Terrier* | 0 0.00 | 1 0.74 | 1 |
| *Terrier Mix* | 0 0.00 | 3 2.22 | 3 |
| *Wheaten Terrier* | 0 0.00 | 1 0.74 | 1 |
| *Total* | 37 | 135 | 172 |
| *Frequency Missing = 2* | | | |

| *Table of breed5 by Epidural* | | | |
| --- | --- | --- | --- |
| *breed5* | *Epidural(Epidural)* | | |
| *Frequency Col Pct* | *No* | *Yes* | *Total* |
| *Golden Retriever* | 1 2.70 | 12 8.76 | 13 |
| *Hound Mix* | 0 0.00 | 5 3.65 | 5 |
| *Labrador Mix* | 0 0.00 | 12 8.76 | 12 |
| *Labrador Retriever* | 8 21.62 | 16 11.68 | 24 |
| *Mixed Breed* | 6 16.22 | 15 10.95 | 21 |
| *Other LT5* | 15 40.54 | 50 36.50 | 65 |
| *Pit Bull Mix* | 3 8.11 | 15 10.95 | 18 |
| *Pit Bull Terrier* | 2 5.41 | 6 4.38 | 8 |
| *Rottweiler* | 2 5.41 | 6 4.38 | 8 |
| *Total* | 37 | 137 | 174 |

| *Table of breed5 by Epidural* | | | |
| --- | --- | --- | --- |
| *breed5* | *Epidural(Epidural)* | | |
| *Frequency Col Pct* | *No* | *Yes* | *Total* |
| *Golden Retriever* | 1 2.70 | 12 8.76 | 13 |
| *Hound Mix* | 0 0.00 | 5 3.65 | 5 |
| *Labrador Mix* | 0 0.00 | 12 8.76 | 12 |
| *Labrador Retriever* | 8 21.62 | 16 11.68 | 24 |
| *Mixed Breed* | 6 16.22 | 15 10.95 | 21 |
| *Other LT5* | 15 40.54 | 50 36.50 | 65 |
| *Pit Bull Mix* | 3 8.11 | 15 10.95 | 18 |
| *Pit Bull Terrier* | 2 5.41 | 6 4.38 | 8 |
| *Rottweiler* | 2 5.41 | 6 4.38 | 8 |
| *Total* | 37 | 137 | 174 |

| *Table of Sex by Epidural* | | | |
| --- | --- | --- | --- |
| *Sex(Sex)* | *Epidural(Epidural)* | | |
| *Frequency Col Pct* | *No* | *Yes* | *Total* |
| *FI* | 1 2.70 | 0 0.00 | 1 |
| *FS* | 17 45.95 | 68 51.13 | 85 |
| *MI* | 0 0.00 | 2 1.50 | 2 |
| *MN* | 19 51.35 | 63 47.37 | 82 |
| *Total* | 37 | 133 | 170 |
| *Frequency Missing = 4* | | | |

| *Table of Limb by Epidural* | | | |
| --- | --- | --- | --- |
| *Limb(Limb)* | *Epidural(Epidural)* | | |
| *Frequency Col Pct* | *No* | *Yes* | *Total* |
| *Left* | 20 54.05 | 64 47.06 | 84 |
| *Right* | 17 45.95 | 72 52.94 | 89 |
| *Total* | 37 | 136 | 173 |
| *Frequency Missing = 1* | | | |

| *Table of BCS by Epidural* | | | |
| --- | --- | --- | --- |
| *BCS(BCS)* | *Epidural(Epidural)* | | |
| *Frequency Col Pct* | *No* | *Yes* | *Total* |
| *2* | 0 0.00 | 1 0.74 | 1 |
| *3* | 29 78.38 | 124 91.18 | 153 |
| *4* | 8 21.62 | 11 8.09 | 19 |
| *Total* | 37 | 136 | 173 |
| *Frequency Missing = 1* | | | |
